# High tibial osteotomy: closed wedge versus combined wedge osteotomy

**DOI:** 10.1186/1471-2474-15-124

**Published:** 2014-04-11

**Authors:** Maarten R Huizinga, Reinoud W Brouwer, Tom M van Raaij

**Affiliations:** 1 Department of Orthopedics, Martini Hospital Groningen, Van Swietenplein 1, 9728 NT, Groningen, The Netherlands

**Keywords:** Knee, Tibial, Osteotomy, Osteoarthritis, Combined, Medial, Unicompartmental, Proximal

## Abstract

**Background:**

High tibial osteotomy is a common procedure to treat symptomatic osteoarthritis of the medial compartment of the knee with varus alignment. This is achieved by overcorrecting the varus alignment to 2-6° of valgus. Various high tibial osteotomy techniques are currently used to this end. Common procedures are medial opening wedge and lateral closing wedge tibial osteotomies. The lateral closing wedge technique is a primary stable correction with a high rate of consolidation, but has the disadvantage of bone loss and change in tibial condylar offset. The medial opening wedge technique does not result in any bone loss but needs to be fixated with a plate and may cause tibial slope and medial collateral ligament tightening. A relatively new technique, the combined valgus high tibial osteotomy, claims to include the advantages of both techniques without bone loss. Aim of this prospective randomized trial is to compare the lateral closing wedge with the combined wedge osteotomy in patients with symptomatic varus osteoarthritis of the knee.

**Methods/design:**

A group of 110 patients with osteoarthritis of the medial compartment of the knee with 6-12° varus malalignment over 18 years of age are recruited to participate a randomized controlled trial. Patients are randomized to undergo a high tibial osteotomy, with either a lateral closing wedge technique or a combined wedge osteotomy technique. Primary outcome measure is achievement of an overcorrection of 4° valgus after one year of surgery, assessed by measuring the hip-knee-ankle angle. Secondary objectives are radiological scores and anatomical changes after high tibial osteotomy; pain, functional scores and quality of life will also be compared.

**Discussion:**

Combined high tibial osteotomy modification avoids metaphyseal tibial bone loss, decreasing transposition of the tibial condyle and shortening of the patellar tendon after osteotomy, even in case of great correction. The clinical results of the combined wedge osteotomy technique are very promising. Hypothesis is that the combined wedge osteotomy technique will achieve more accurate overcorrection of varus malalignment with fewer anatomical changes of the proximal tibia after one year.

**Trial registration:**

Dutch Trial Registry (Netherlands trial register): NTR3898.

## Background

Knee osteoarthritis (OA) is the most common joint disorder, and the lifetime risk of developing symptomatic knee OA has been estimated to be around 45% [1,2]. Many patients present with unicompartmental knee OA, which is generally caused by a mechanical problem and may lead to knee malalignment [3]. Knee malalignment is a determinant of load distribution, and is associated with OA progression [4].

Alterations in the mechanical stresses on the knee joint tissues improves symptoms and reduces the risk of developing radiographic knee OA [5]. Valgus-producing high tibial osteotomy (HTO) does correct varus malalignment and is an accepted treatment for medial unicompartmental knee OA in active patients [6]. Aim of the HTO procedure is to offload the medial compartment by overcorrecting the varus malalignment to 2-6° of valgus [7-11].

The two most commonly used surgical techniques for HTO are lateral closing wedge HTO (LCW) and medial opening wedge HTO (MOW) with internal fixation, which has gained popularity in recent years. In both techniques the goal is to achieve and maintain adequate correction of malalignment of the knee joint. In the lateral closing wedge technique a wedge of bone with the base on the lateral side is removed from the proximal tibia, producing valgus. A fibular osteotomy or release of the proximal tibiofibular joint is necessary. After LCW, lateral tibial metaphyseal bone loss may occur and can lead to considerable lateral overhang of the tibial plateau, producing changes in tibiocondylar offset. MOW does not cause any bone loss but needs stable plate fixation with possible tibial slope changes, medial collateral ligament tightening and patella baja [12]. There are no differences between LCW and MOW in frequency and type of complications [13].

A relatively new HTO technique, the combined valgus-producing high tibial osteotomy (CWO), claims to include the advantages of both the LCW and the MOW techniques for HTO. This HTO modification avoids metaphyseal tibial bone loss, decreasing transposition of the tibial condyle and shortening of the patellar tendon after osteotomy, even in cases of great correction [14]. The aim of this prospective randomized trial (RCT) is to compare LCW with CWO in patients eligible for HTO who have varus alignment up to 12°. Hypothesis is that the CWO technique will achieve more accurate overcorrection of varus malalignment with fewer anatomical changes of the proximal tibia after one year.

## Methods/design

### Study design

The study design is a randomized controlled trial. Patients will be allocated to two groups: HTO with CWO technique or HTO with LCW technique. The study will be conducted at the Orthopedic Department of Martini Hospital in Groningen. The study design, procedures, protocols and informed consent are approved by the local Medical Ethical Committee. The trial is registered in the Netherlands Trial Registry (NTR3898).

### Study population

Patients visiting the outpatient clinic of the Orthopedic Department who have an indication to undergo HTO because of osteoarthritis of the medial compartment of the knee with varus alignment will be included if they meet the following criteria: radiologically confirmed medial compartment osteoarthritis, medial joint pain, 6-12° varus alignment and age 18 and older. Exclusion criteria are: symptomatic osteoarthritis of the lateral compartment, rheumatoid arthritis, range of motion of the knee joint under 100°, flexion contracture more than 10°, grade 3 collateral laxity (Insall), history of fracture or previous open operation of the lower extremity, mental incapacity, or inability to fill in the questionnaires in Dutch.

### Interventions

After randomization patients will undergo either a CWO HTO or a LCW HTO; all patients will be operated on by one of two orthopedic surgeons, both experienced in applying the two techniques so there will be no bias. Preoperative the wedge-correction is calculated aiming on 4° overcorrection.

#### Combined wedge osteotomy (CWO)

For the combined wedge HTO, instrumentation of Allopro (Zimmer; Winterthur, Switzerland) will be used. The common peroneal nerve will be exposed and snared with a nerve band. Next, the anterior part of the proximal fibular head (anterior part of the proximal tibiofibular syndesmosis) will be resected. The proximal osteotomy is performed using an oscillating saw up to the center of the tibial head, 2 cm distally from the joint line. After positioning the aiming device in the first osteotomy, a second distal osteotomy is made, which results in a laterally-based bone wedge. The bone wedge is removed. The distal part of the tibia is placed in valgus position, so the lateral part of the osteotomy closes and the medial part of the osteotomy opens. The center of the valgisation is the center of the tibial head. The half bone wedge is placed back in the gap opened on the medial side. The osteotomy is secured with staples, two on the lateral side and one on the medial side. At the end of the procedure a fasciotomy of the anterior compartment will be performed to prevent compartment syndrome.

#### Lateral closing wedge osteotomy (LCW)

For the lateral closing wedge HTO, instrumentation of Allopro (Zimmer; Winterthur, Switzerland) will be used. The common peroneal nerve will be exposed and snared with a nerve band. Next, the anterior part of the proximal fibular head (anterior part of the proximal tibiofibular syndesmosis) will be resected. The osteotomy will be made and the wedge taken out. The osteotomy will be fixated with two staples. At the end of the procedure a fasciotomy of the anterior compartment will be performed to prevent compartment syndrome.

The postoperative treatment for both HTO techniques consists of a pressure bandage for 24 hours and cast immobilization until removal of the stitches, followed by flexion-extension brace protection and 50% weight bearing for 6 weeks. After this period, weight bearing can be built up to 100% based on the pain sensation of the patient, brace use can be reduced to no brace, and physical therapy will be started.

### Main study parameter/endpoint

Primary outcome measure is the accuracy and preservation correction of the technique, defined by achievement of an overcorrection of 4° valgus one year after surgery as measured by the hip-knee-ankle (HKA) angle. The continuous differences in achievement of a valgus overcorrection and the deviation from 4° valgus will be determined, as well as dichotomous outcome and achievement of a 0-6° valgus alignment .

### Secondary study parameters/endpoints

Secondary outcome measures are anatomical changes due to HTO and pain and function scores. Parameters are tibial slope (Moore-Harvey and Dejour-Bonin); patellar height (Insall-Salvati and Caton Index) and difference in leg length (cm) one year after surgery; and pain severity (VAS; range 0–10), knee function and quality of life (KOOS), and walking distance (km) at six weeks, three months, six months and one year after surgery.

### Other study parameters

Potential confounding parameters, such as opposite cortex fractures, demographic data, length, weight and BMI will be recorded. Adverse events like re-operations, including hardware removal, will also be recorded**.**

### Randomization, blinding and treatment allocation

For randomization a restricted randomization/blocked randomization (2×10 patients in one block) method will be used. The same number of patients will be allocated to each surgical treatment group. Two random allocation sets (for the CWO and the LCW) will be generated by a computer. These allocations are then sealed in consecutively numbered opaque envelopes. Once the patient has given consent to be included in the trial, the HTO technique (CWO or LCW) is randomly assigned by opening the next sealed envelope.

### Study procedures

Preoperatively as well as six weeks, six months and one year postoperatively, patients will visit the outpatient clinic. Routine physical examination will be performed by the orthopedic surgeon.

Primary outcome measure–achievement of an overcorrection of 4° valgus alignment / hip-knee-ankle angle (HKA, in degrees)–will be measured after one year on a whole-leg radiograph (WLR) standing on one leg. The continuous differences in achievement of a valgus overcorrection and the deviation from 4° valgus are determined. A dichotomous outcome is achievement of a 2-6° valgus alignment.

Secondary outcome measures, posterior tibial slope (Moore-Harvey and Dejour-Bonin), patellar height (Insall-Salvati and Caton Index) and difference in leg length (cm) one year after surgery will be obtained. Pain severity (VAS; range 0–10), knee function and quality of life (KOOS), and walking distance (km) six weeks, three months, six months and one year after surgery will also be obtained.

### Sample size calculation

The sample size was calculated based on an expected increase of the success rate from 60% in the closed wedge HTO to 85% in the combined wedge HTO. A successful operative result is defined as achievement of a 2-6° valgus alignment. To detect such a difference with α= 0.05 and a power of 80%, we need to include 50 patients in each study group. To adjust for possible loss to follow-up, a total of 110 patients will be included in the study.

### Statistics

All data will be analyzed according to an intention-to-treat principle, implying that all patients who are randomized will be included in the analyses, and that they will be analyzed according to the group to which they were allocated. For those patients who will be lost to follow-up or will be re-operated during follow-up, the last available measurement or the last measurement will be forwarded (Last Value Carried Forward technique).

A multivariable linear regression method will be used to analyze the impact of LCW versus CWO HTO on postoperative alignment, posterior tibial slope, patellar height, leg length, VAS, KOOS, walking distance and patients with adverse events at the one-year follow-up. A multivariable logistic regression method will be used for the dichotomous outcome measures. Gender, age and baseline values for HKA angle, VAS knee, KOOS, walking distance, medial osteoarthritis more than joint space loss alone, and concurrent OA of the lateral compartment will be considered as possible confounders and are included in the regression models only if they change the relationship between dependent variable and type of HTO by at least 10%. The SPSS program will be used for the statistical analyses and a p-value of 0.05 is considered statistically significant.

## Discussion

Although HTO remains quite successful after 10 years, OA with associated symptoms will progress. Some patients may require total knee replacement (TKR) [15]. Success of TKR for knee OA is well-established, and about 85% of patients are satisfied with the surgical outcome [16], therefore when considering HTO in the early treatment of symptomatic medial compartmental OA subsequent TKA should not be compromised. A randomized clinical trial showed significantly more patellar descent and tibial slope increase after MOW compared to the LCW technique [17]. This may cause exposure and patellar eversion problems during knee replacement that may compromise precision and accuracy of TKR [18]. The advantage of MOW is preservation of bone stock with tensioning of the medial collateral ligament. This may result in a more conservative amount of bone removed during knee joint replacement, so joint line elevation by using a thicker-than-desired tibial component in balancing the ligaments is less likely. Furthermore, unlike LCW, the relative position of the medullary canal is not altered. This may facilitate tibial component placement with intramedullary guidance.

An operative complication that may result in an adverse effect is intraoperative fracturing of the opposite cortex. Avoidance of opposite cortex fracture can be difficult though, and is generally limited by the angular size of the wedge. Opposite cortical fracture occurs often during the LCW technique [15]. A fractured medial cortex in LCW may lead to progressive movement of the distal tibia into a varus position [19]. In case of the MOW, instability at the MOW site due to disruption of the lateral cortical hinge potentially results in displacement of the osteotomy and may contribute to recurrent varus deformity [20]. Newer angular stable plate systems with locking screws seem to avoid this problem. Loss of correction may lead to a suboptimal result [11,21,22]. Still, HTO is considered a very successful surgical procedure with low complication rates. For instance, non-union in HTO is very rare, especially in LCW, and delayed union is seen in about 3-4% [23,24].

A Cochrane review showed no evidence as to whether LCW or MOW is more effective in the treatment of symptomatic medial knee OA. A relatively new HTO technique, the combined valgus-producing high tibial osteotomy (CWO), claims to include the advantages of both the LCW and MOW techniques for HTO. This HTO modification avoids metaphyseal tibial bone loss, decreasing transposition of the tibial condyle and shortening of the patellar tendon after osteotomy, even in cases of great correction [14]. Both the LCW and CWO techniques have been commonly used for HTO at the Orthopedics Department of Martini Hospital for years now. The clinical results of the CWO technique are very promising. So far there is little scientific evidence on its effectiveness though. Hence the aim of this prospective randomized trial (RCT) is to compare LCW with CWO in patients eligible for HTO.

## Abbreviations

OA: Osteoarthritis; HTO: High tibial osteotomy; LCW: Lateral closing wedge HTO; MOW: Medial opening wedge HTO; CWO: Combined wedge osteotomy; RCT: Randomized controlled trial; WLR: Whole-leg radiograph; HKA: Hip-knee-ankle angle; TKR: Total knee replacement.

## Competing interests

The authors will not receive any reimbursements, fees or salary for conducting the study.

## Authors’ contributions

MH, RB and TR originated the idea for the study, contributed to its design and developed the intervention protocol. MH is responsible for the data collection. MH and RB drafted the manuscript. All authors read, edited and approved the final manuscript.

## Pre-publication history

The pre-publication history for this paper can be accessed here:

http://www.biomedcentral.com/1471-2474/15/124/prepub
